# Case Report: A case of Fraser syndrome 2 in a Chinese fetus caused by novel compound heterozygous variants in the *FREM2* gene

**DOI:** 10.3389/fmed.2025.1705708

**Published:** 2025-12-04

**Authors:** Weidong Wei, Yinan Fan, Jiaqi Song, Yedan Lou, Tao Zhang, Hua Yuan, Jieyuan Jin, Sinan Zhang, Xin Jin

**Affiliations:** 1 Shaoxing Maternity and Child Health Care Hospital, Shaoxing, Zhejiang, China; 2 Obstetrics and Gynecology Hospital of Shaoxing University, Shaoxing, Zhejiang, China; 3 Shaoxing University, Shaoxing, Zhejiang, China; 4 Department of Respiratory and Critical Care Medicine, Shanghai Changhai Hospital, The First Affiliated Hospital of Naval Military Medical University, Shanghai, China

**Keywords:** Fraser syndrome 2, *FREM2*, compound heterozygous, prenatal diagnosis, Trio-WES

## Abstract

**Background:**

Fraser syndrome (FS) is an autosomal recessive inherited malformation disorder characterized by cryptophthalmos, syndactyly, and abnormalities of the respiratory and urogenital tracts. Variants in the FRAS1-related extracellular matrix 2 (FREM2) gene are the major genetic cause. However, clinical diagnosis remains challenging due to phenotypic heterogeneity.

**Methods:**

A 24-week pregnant woman came to our hospital for genetic diagnosis. Ultrasound examination showed bilateral renal agenesis or dysplasia, absence of the bladder, and almost oligohydramnios. Trio whole-exome sequencing (Trio-WES) identified two novel compound heterozygous variants in the fetal FREM2 gene: a maternal, frameshift variant, c.5908_5909del, p.Leu1970ValfsTer33, and a paternal, nonsense variant, c.7881C>G,p.Tyr2627Ter.

**Conclusion:**

We report a rare case of Fraser syndrome 2 caused by compound heterozygous mutations in the FREM2 gene. Our findings expanded the *FREM2* genotypic spectrum and demonstrated the significance of Trio-WES in the prenatal diagnosis of recessive disorders.

## 1 Introduction

Fraser syndrome (FS) 2 (OMIM#617666) is an autosomal recessive genetic disorder characterized by cryptophthalmos, syndactyly, and abnormalities of the respiratory and genitourinary tracts (1). The clinical phenotype may also include a low-set hairline, hypoplastic alae nasi, small mouth, short thorax, abdominal distension, ambiguous genitalia, vaginal atresia, renal agenesis, renal hypoplasia, ureteral agenesis, direct connection of the ureter to the vagina, and bladder agenesis.

Fraser syndrome 2 is a very rare disorder with approximately 150 cases reported to date. The genetic background of this disease is usually associated with *FRAS1*, *FREM2*, and *GRIP1*. *FRAS1* is a gene that is involved in skin epithelial morphogenesis during early development (2). *FREM2* is located on chromosome 13, and *GRIP1* is located on chromosome 12. The main function of the *FREM2* gene is to participate in the formation and maintenance of the extracellular matrix. The extracellular matrix is a supporting structure that surrounds cells and significantly influences cell morphology, function, and interactions. The FREM2-encoded protein maintain normal tissue structure by interacting with other components in the extracellular matrix (3). These genes are crucial for cell adhesion and extracellular matrix structure during embryonic development, and variants can also lead to abnormal embryonic development.

Prenatal diagnosis of Fraser syndrome 2 can be performed by ultrasound examination; however, due to the great variety of possible malformations, the diagnosis will remain uncertain in most cases (4). The primary diagnostic criteria include cryptophthalmos, cutaneous syndactyly, genitourinary malformations, and laryngotracheal abnormalities. In response to this situation, we need to conduct further gene sequencing. Treatment and prognosis of FS usually involve dealing with the severity of cerebral, pulmonary, laryngeal, and renal malformations (5). Treating this disease requires multidisciplinary collaboration, including interventions such as total auricular reconstruction in otolaryngology and symblepharon release in ophthalmology.

In this study, we report a fetus with Fraser syndrome 2 caused by two variants in the FREM2 gene. Trio whole-exome sequencing (Trio-WES) revealed two novel variants not previously included in the ClinVar database: c.5908_5909del, p.Leu1970ValfsTer33 and c.7881C>G, p.Tyr2627Ter. Pedigree testing confirmed that the former variant originated from the mother, while the latter variant was inherited from the father. These findings demonstrate the importance of prenatal diagnosis. The genetic sites of these two variants were identified initially, and we hope this novel discovery will help geneticists gain further insights into Fraser syndrome 2.

## 2 Materials and methods

### 2.1 Sample collection

This study was approved by the Institutional Ethics Committee of Shaoxing Maternity and Child Health Care Hospital. An informed consent document for participation and publication was signed by the family members. With parental consent, we collected venous blood samples from both parents, and fetal DNA was extracted from the amniotic fluid.

### 2.2 Trio whole-exome sequencing

Genomic DNA was extracted from blood samples of the patient and both parents using a DNEasy Blood and Tissue Kit (Qiagen, Hilden, Germany), according to the manufacturer’s procedures. For Trio-WES, the genomic DNAs were enriched for coding exons using Agilent SureSelect Low Input Reagent Kit and sequenced on the Illumina HiSeq X Ten platform. The sequencing data captured 99.89% of coding regions across 37,837,472 base pairs across 20,858 genes. The average sequence depth was 144.446X, with 97.91% of targeted regions achieving a depth of ≥20X.

### 2.3 Data analysis

Quality assessment of raw sequencing data was performed using fastp software, and the low-quality reads, as well as those contaminated by adapters, were removed. The filtered data were aligned with the human hg19 reference genome using BWA software, and then the capture efficiency was evaluated. Then, the single-nucleotide variants (SNVs) and indels in the genome were analyzed using GATK software. Subsequently, the identified SNV and Indel were filtered using population databases, including 1,000 Genomes (1,000 human genome dataset), Genome Aggregation Database dataset (gnomAD) 2.1.1, and the Exome Aggregation Consortium dataset (ExAC). The pathogenicity of missense variants and splicing variants was predicted using the dbNSFP database. Reported variants were used in databases including OMIM, HGMD, and ClinVar. All variants were classified according to American College of Medical Genetics and Genomics (ACMG) genetic variant classification standards and guidelines. Finally, all potentially pathogenic sites were validated by Sanger sequencing.

Similar mitochondrial genome sequencing methods were used, and the obtained data were aligned with the mitochondrial reference genome NC_012920.1. The mitimpact29, HmtVar, and tRNA databases were used to predict the pathogenicity of missense variants and splicing variants. The ClinVar database was used to screen for reported variants.

## 3 Results

### 3.1 Clinical features

**Figure 1:**
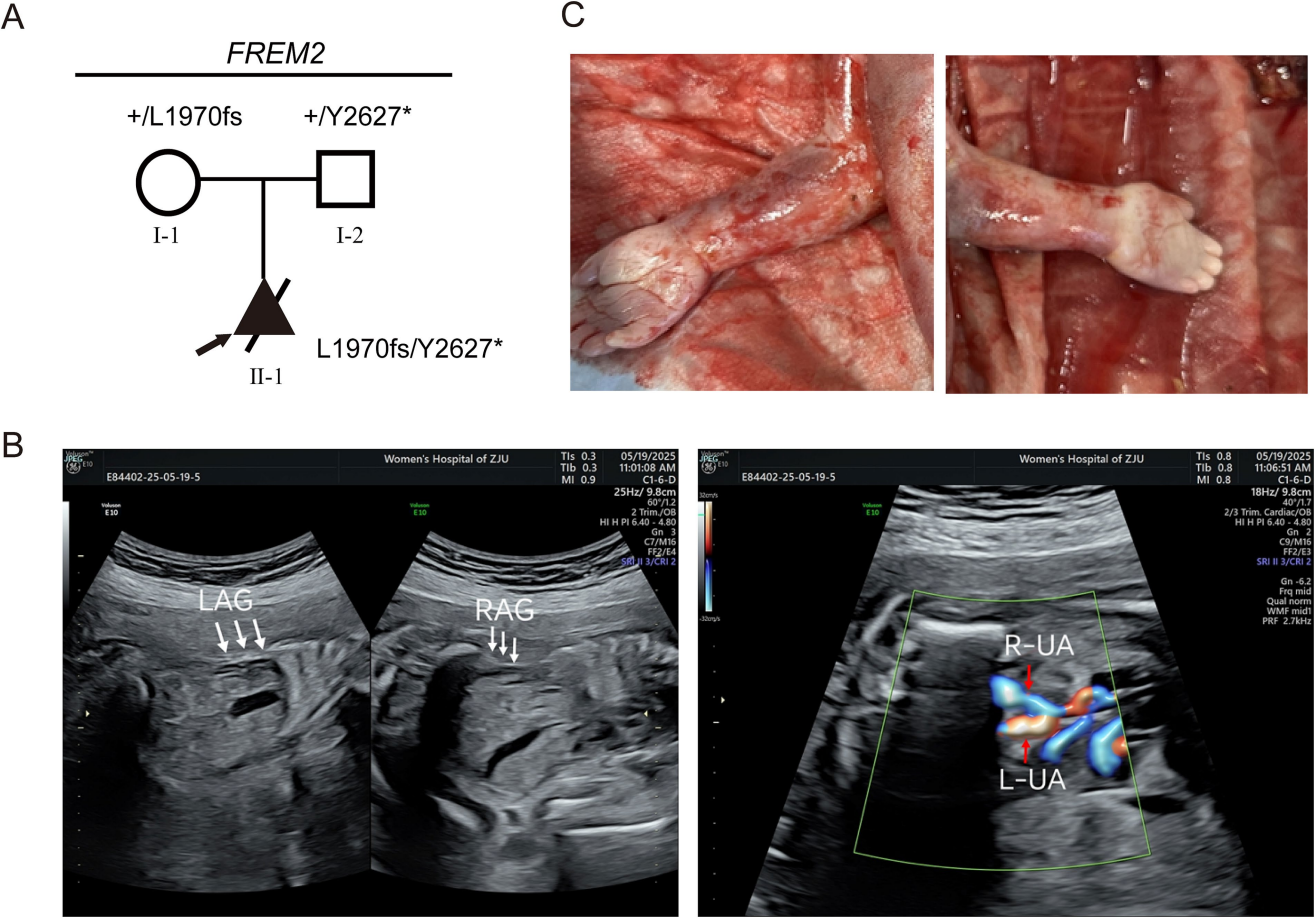
Clinical phenotypes of patients in the Fraser syndrome 2 pedigree associated with *FREM2* variants. **(A)** Pedigree analysis of the presented family. The frameshift variant (c.5908_5909del, p.Leu1970ValfsTer33) occurred in a heterozygous state in the mother (I-1). The nonsense variant (c.7881C>G, p.Tyr2627Ter) occurred in a heterozygous state in the father (I-2). The compound heterozygous variants occurred in the fetus (II-1). Round symbols indicate female individuals; square symbols indicate male individuals; the triangle symbols indicate a fetus; arrow marks indicate the proband. The plus signs denote the reference allele. **(B)** No renal echoes were visualized in the bilateral renal areas adjacent to the fetal spine, and the lying-down adrenal gland sign was observed (indicated by the white arrow). LAG, left adrenal gland; RAG, right adrenal gland. No bladder echo was seen between the left and right umbilical arteries on the transverse section of the fetal lower abdomen. L-UA, left umbilical artery, R-UA, right umbilical artery. **(C)** Clinical photographs of the proband show cutaneous symbrachydactyly of the hands.

At 24 weeks of pregnancy, an ultrasound showed that the fetus had bilateral renal agenesis or dysplasia, with no visible bladder and almost no amniotic fluid. No renal echoes were visualized in the bilateral renal areas adjacent to the fetal spine, and the lying-down adrenal gland sign was observed (indicated by the white arrow). No bladder echo was seen between the left and right umbilical arteries on the transverse section of the fetal lower abdomen (Figure 1). Chromosomal testing confirmed a male fetus. The mother was a 28-year-old gravida 1, para 0, and the father was 31 years old. There was no documented history of consanguinity between the parents. This was their first pregnancy, and she conceived spontaneously.

Parents have no relevant genetic history, and the fetal genetic inheritance was analyzed using STR testing.

### 3.2 Identification of a novel variant in *FREM2* gene

**Figure 2:**
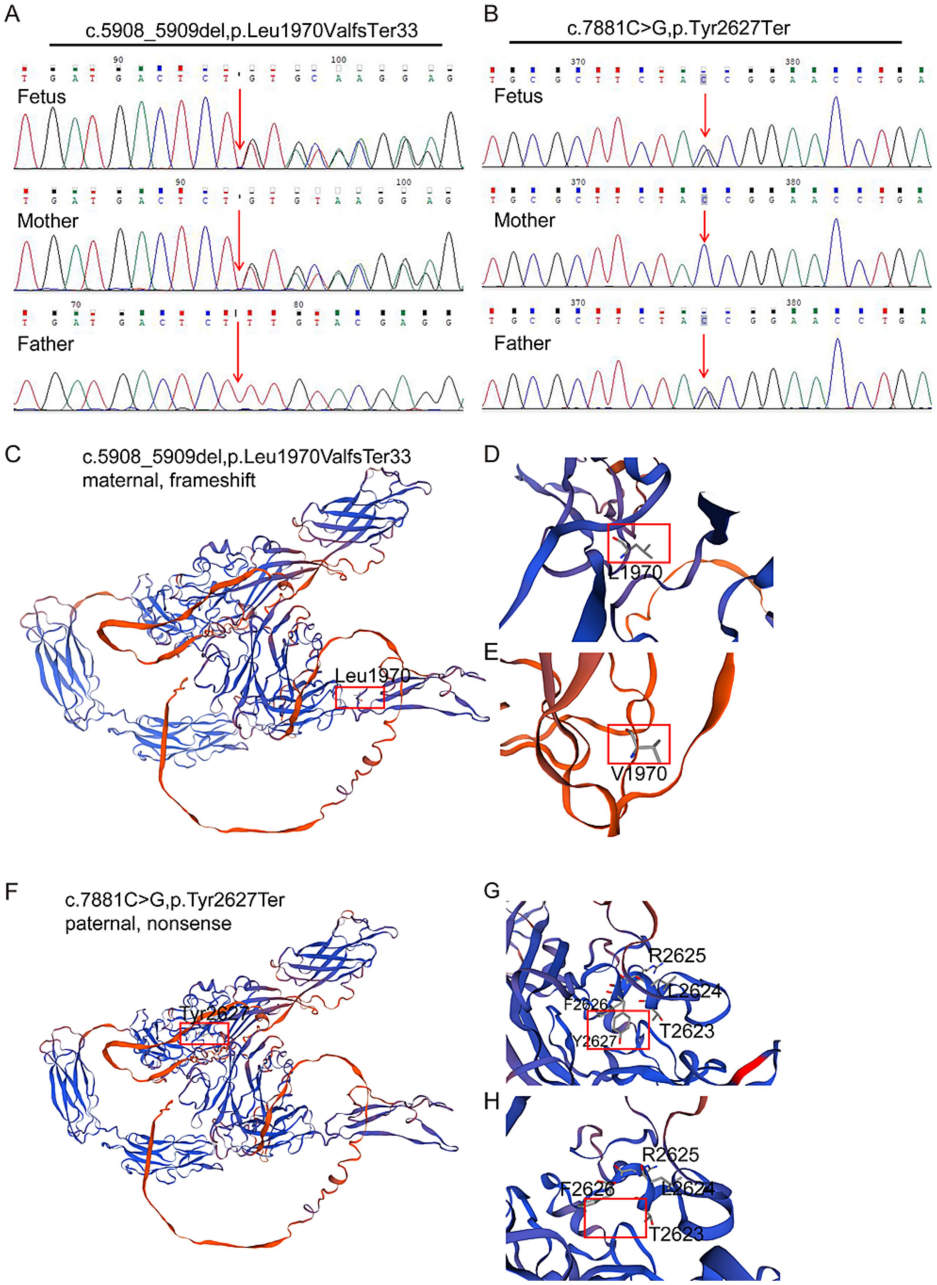
Characterization of identified novel mutations in *FREM2*. **(A,B)** Sanger sequencing verification of biallelic *FREM2* variants in the pedigree. Variants are denoted in red font. The compound heterozygous variants of *FREM2* were identified in the fetus. **(C,D)** Part of the three-dimensional structure of the wild-type *FREM2*; and **(E)** pathogenic variant type (c.5908_5909del; p.Leu1970ValfsTer33). **(F,G)** Part of the three-dimensional structure of the wild type *FREM2*; and **(H)** likely pathogenic variant type (c.7881C>G; p.Tyr2627Ter).

Trio-WES was performed to detect the presence of any variant(s) in the related disease-causing genes. The sequencing was performed using capture high-throughput chip technology, and over 20,000 genes were detected from the human genome. The Sanger sequence was used to verify the variants. The results showed that two variants exist in the *FREM2* gene, namely c.5908_5909del (PVS1 + PM2 + PM3) and c.7881C>G (PVS1 + PM2 + PM3), classified as pathogenic variants according to the American College of Medical Genetics and Genomics (ACMG) criteria. The former is a frameshift variant caused by the deletion of nucleotides 5,908 to 5,909 in the coding region, resulting in a change from leucine to valine at amino acid position 1970 and creating a new reading frame that terminates at codon 33 downstream (PVS1). This variant results in a premature termination of the coding protein sequence. The latter is a nonsense variant, which is a cytosine to guanine substitution at nucleotide position 7,881, resulting in a premature stop codon at amino acid position 2,627 (PVS1). This variant also causes the early termination of the coding protein sequence. Subsequently, DNA was extracted from the affected fetus and blood samples were collected from his parents, and the variants were verified by Sanger sequencing, which confirmed that the variant (*FREM2* c.5908_5909del, p.Leu1970ValfsTer33) was inherited from the mother and the other variant (*FREM2* c.7881C>G,p.Tyr2627Ter) was inherited from his father (PM3). This is the major cause of the fetus’s illness (Figures 2A,B). Neither of the two mutations has been reported in databases such as gnomAD (PM2).

### 3.3 Structural analysis of FREM2 protein variants identified in the fetus

To investigate the impact of these variants on protein structure, the three-dimensional structures of the wild-type and mutant FREM2 proteins were analyzed. For the maternal frameshift variant c.5908_5909del (p.Leu1970ValfsTer33) (Figures 2C–E), the deletion leads to a shift in the reading frame, causing amino acid 1970 to change from leucine (Leu, shown in Figure 2D) to valine (Val, shown in Figure 2E), and subsequently causing premature termination. This results in a truncated protein structure, resulting from the loss of a C-terminal region beyond the new termination site. The alteration at position 1970 and the truncation disrupt the normal folding and spatial arrangement of the protein, particularly affecting the domain where Leu1970 is located, which may impair interactions with other molecules.

Regarding the paternal nonsense variant c.7881C>G (p.Tyr2627Ter) (Figures 2F–H), the substitution introduces a premature stop codon at amino acid 2,627. In the wild-type protein, the region around Tyr2627 (Figure 2F) has a specific conformation to neighboring residues such as R2625, F2626, L2624, and T2623. In the mutant protein (Figure 2H), the truncation removes Tyr2627 and the subsequent amino acids, leading to a loss of this structural segment and disrupting the overall protein architecture. Both variants result in significant structural abnormalities of the FREM2 protein, which is likely to compromise its normal function and contribute to the observed phenotypic abnormalities.

### 3.4 Predicted protein interaction network of FREM2

**Figure 3:**
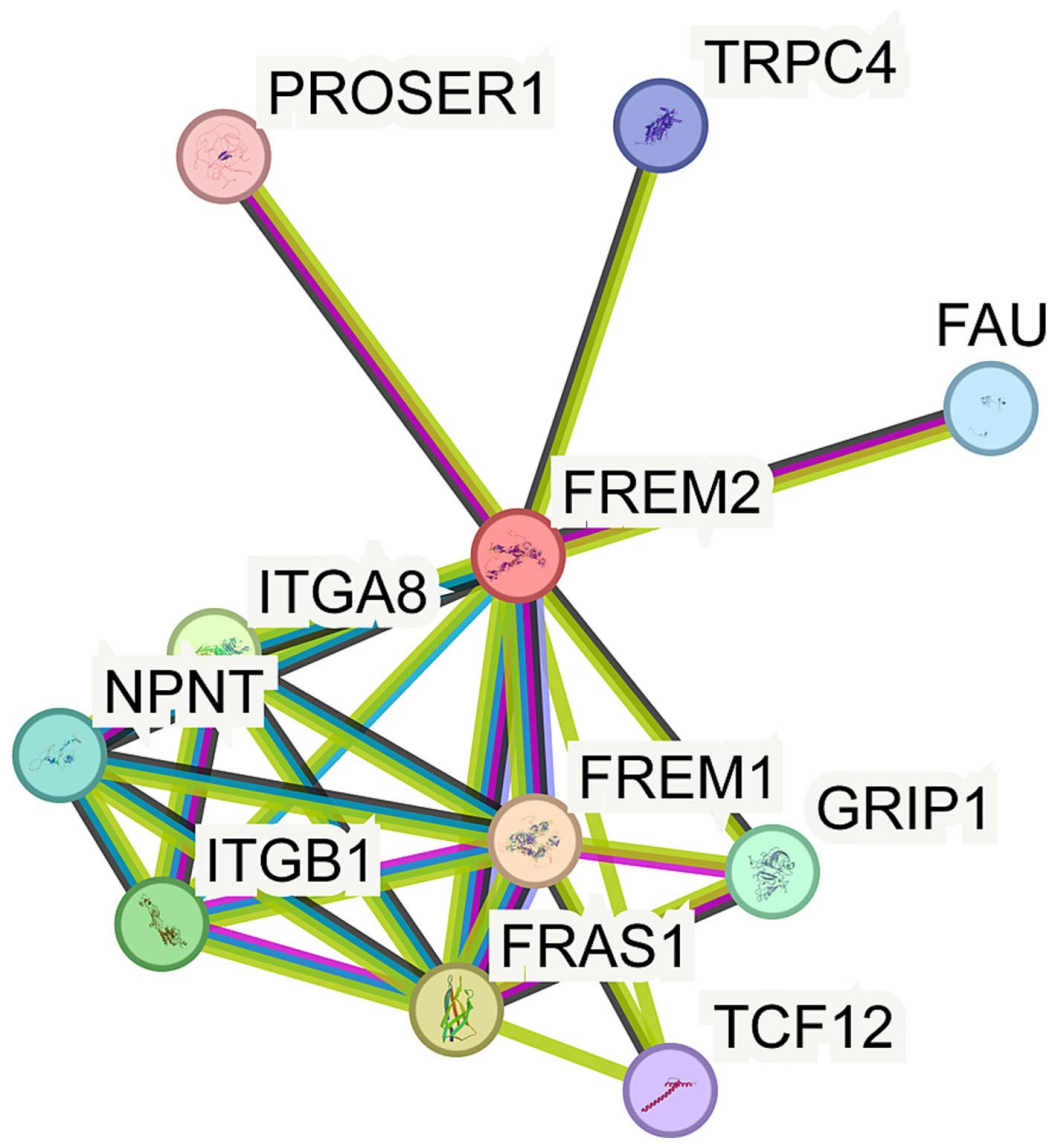
Human protein interactors as a candidate for FREM2 collected from the previously reported experimental methods. The network was created using STRING.

To explore the potential interacting proteins of FREM2 and gain insights into its functional network, we utilized the STRING database for protein–protein interaction (PPI) analysis (Figure 3). FREM2 (red node) was predicted to interact with a range of proteins. These include FRAS1, FREM1, and GRIP1, which are known to be associated with Fraser syndrome, suggesting a coordinated role in the biological processes underlying this disorder. Additionally, interactions were identified with proteins such as ITGA8, ITGB1, NPNT, PROSER1, TRPC4, FAU, and TCF12. The diverse array of interacting proteins implies that FREM2 may participate in multiple cellular pathways, including those related to cell adhesion, extracellular matrix organization, and signal transduction. This PPI network provides a foundation for further investigating the molecular mechanisms by which FREM2 variants lead to the phenotypic abnormalities observed in Fraser syndrome.

## 4 Discussion

Fraser syndrome 2 is a rare recessive genetic disorder with limited medical research. This article reports a case of Fraser syndrome 2 caused by compound heterozygous variants in the *FREM2* gene, hoping to further enrich the understanding and research of this disease.

In this case, the fetus inherited different variants in the same gene (*FREM2*) from the parents. One is c.5908_5909del, p.Leu1970ValfsTer33 (a frameshift variant caused by the deletion of nucleotides 5,908 to 5,909 in the coding region, resulting in a change from leucine to valine at amino acid position 1970 and creating a new reading frame that terminates at codon 33 downstream). The other variant is c.7881C >G, p.Tyr2627Ter (a cytosine to guanine substitution at nucleotide position 7,881, resulting in a premature stop codon at amino acid position 2,627), which is a nonsense variant. According to the analysis of the pathogenic variant, both variants can lead to the premature termination of the coding protein sequence, resulting in truncated proteins. Many experiments have demonstrated that the introduction of a stop codon results in a truncated protein, leading to loss of biological function of protein (6). Therefore, in this case, the affected fetus is most likely suffering from Fraser syndrome 2 due to compound heterozygous variants that produce truncated proteins, disrupting the original structure of *FREM2*.

According to existing investigations, FREM2 is associated with immune checkpoints, and its variants may serve as prognostic markers in colorectal cancer patients (7). In another study, researchers focused on a common variant site (c.6499C>T) in three patients and concluded that loss-of-function variants in the *FREM2* gene can disrupt eye morphology (8). Cryptophthalmos is a rare congenital disease that is also one of the characteristic features of Fraser syndrome 2. In recent years, cases have been discovered where variants in the *FREM2* gene have led to premature aging and loss of normal function in fetuses (9). Some patients have received assistance based on NGS-based SNP haplotyping to select embryos that meet the requirements and have successfully given birth to a healthy baby through *in vitro* fertilization and embryo transfer (10).

**Figure 4:**
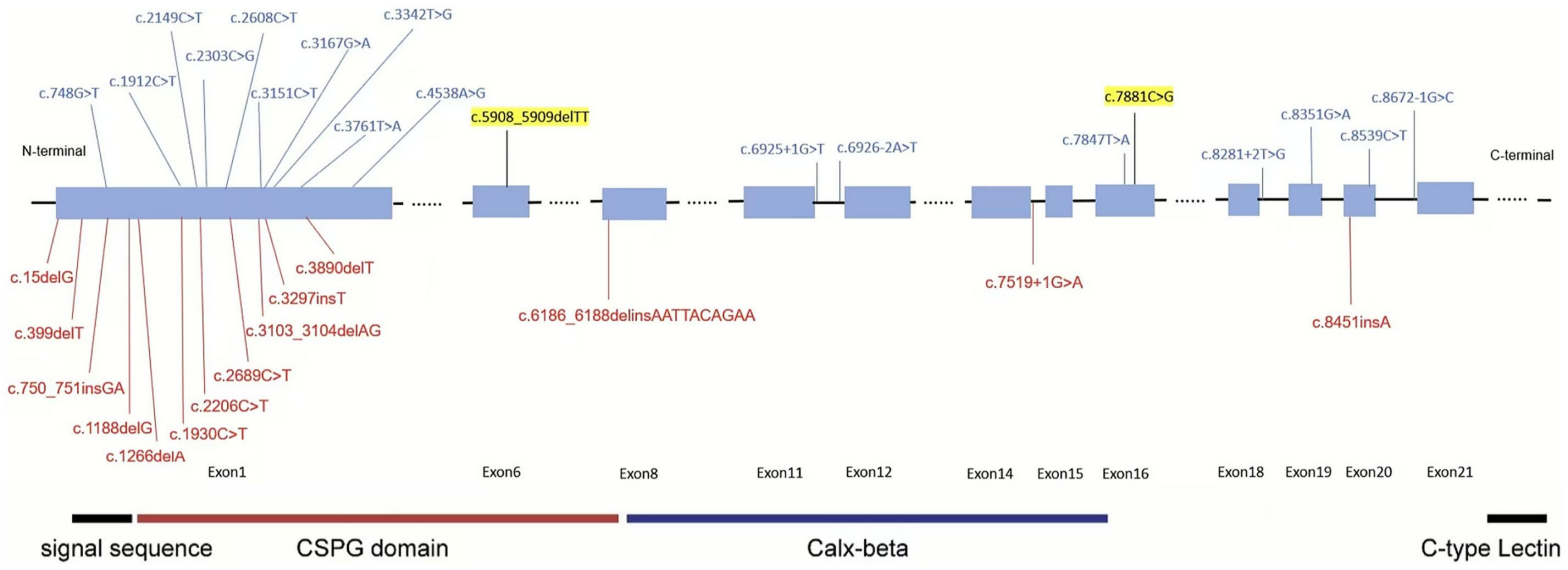
ClinVar-reported variants in the FREM2 gene. The figure displays pathogenic and likely pathogenic variants in the FREM2 gene documented in the ClinVar database, color-coded based on their classification: red for pathogenic (P) and blue for likely pathogenic (LP). Variants highlighted in yellow represent those identified and reported in the current study. The functional domains are highlighted using colored boxes.

To further explore the impacts of *FREM2* gene mutation sites, we have collected and summarized pathogenic and likely pathogenic mutation sites of this gene from the ClinVar database, and creates a diagram (Figure 4). The updated spectrum of ClinVar-reported pathogenic variants in *FREM2* (Figure 4) reveals notable clustering of truncating mutations within the large first exon, which encodes the vast N-terminal portion of the protein. This region encompasses critical structural elements, including the signal peptide and CSPG domains, which are essential for the protein’s secretion and its role in epithelial–mesenchymal interactions. The predominance of loss-of-function variants in this region suggests that complete disruption of the protein’s extracellular function is the primary disease mechanism for FS 2. During the retrieval process, we found that different mutation sites and mutation forms in the *FREM2* gene lead to multiple outcomes. For example, both cases involve compound heterozygous mutations at two different sites of the same gene. However, in this case, the affected fetus mainly showed renal dysplasia, while in the other one, the mutations primarily led to dental and oral deformities (11). The most serious consequence would be the development of cryptophthalmos (8, 12).

From the perspective of protein structure, the two identified variants, c.5908_5909del (p.Leu1970ValfsTer33) and c.7881C>G (p.Tyr2627Ter), both cause severe truncation of the FREM2 protein. The maternal frameshift variant leads to a reading frame shift and premature termination, resulting in the loss of the C-terminal region and disruption of the normal folding and spatial arrangement, especially around the Leu1970 site. The paternal nonsense variant introduces a premature stop codon, removing Tyr2627 and subsequent amino acids, which destroys the overall protein architecture. As mentioned, truncated proteins caused by premature stop codons are generally considered to lose biological function (6), and such structural abnormalities in FREM2 likely impair its ability to perform normal cellular roles, which is a key factor in the development of Fraser syndrome 2 in the fetus.

Regarding the PPI network, FREM2 interacts with several proteins known to be associated with Fraser syndrome, such as FRAS1, FREM1, and GRIP1. This confirms the coordinated role of these proteins in the biological processes underlying the disorder. Additionally, interactions with proteins involved in cell adhesion, extracellular matrix organization, and signal transduction suggest that FREM2 is involved in multiple crucial cellular pathways during embryonic development. The variants in FREM2 not only affect its own structure and function but may also disrupt these interaction networks, further contributing to the complex phenotypic abnormalities observed in Fraser syndrome 2.

Existing studies have also implicated FREM2 in various biological processes and disease contexts. For example, FREM2 is associated with immune checkpoints and may serve as a prognostic marker in colorectal cancer patients (7). Loss-of-function variants in FREM2 can disrupt eye morphology (8), which is relevant to the cryptophthalmos characteristic of Fraser syndrome 2. Moreover, FREM2 variants have been linked to fetal developmental abnormalities, including premature aging and loss of normal function (9), and have implications for assisted reproductive technologies (10). The identification of two novel variants expands the FREM2 variant spectrum and also provides new insights into the genetic basis of Fraser syndrome 2, reinforcing the importance of high-throughput sequencing in uncovering the causes of rare genetic disorders.

In conclusion, this study reports two novel variants in the *FREM2* gene associated with the risk of Fraser syndrome 2. The use of high-throughput sequencing technology provided valuable insights into the cause of variants and disease progression. This new discovery enhances our understanding of FS.

## Data Availability

The original contributions presented in the study are included in the article/supplementary material, further inquiries can be directed to the corresponding author.
